# Genome Sequencing of *Ralstonia solanacearum* Biovar 3, Phylotype I, Strains Rs-09-161 and Rs-10-244, Isolated from Eggplant and Chili in India

**DOI:** 10.1128/genomeA.00323-14

**Published:** 2014-05-29

**Authors:** Raman Ramesh, Sapna Gaitonde, Gauri Achari, Trupti Asolkar, Narendra Pratap Singh, Sebastien Carrere, Stephane Genin, Nemo Peeters

**Affiliations:** a ICAR Research Complex for Goa, Old Goa, Goa, India; b INRA, Laboratoire des Interactions Plantes-Microorganismes (LIPM), Castanet-Tolosan, France; c CNRS, Laboratoire des Interactions Plantes-Microorganismes (LIPM), Castanet-Tolosan, France

## Abstract

*Ralstonia solanacearum* Indian strains Rs-09-161 and Rs-10-244 were isolated from the coastal region of Goa and from the Andaman Islands. We report the draft genome sequences of these representative isolates infecting solanaceous vegetables in India.

## GENOME ANNOUNCEMENT

*Ralstonia solanacearum* is a major plant-pathogenic bacterium that causes bacterial wilt disease in over 400 plant species (1). *R. solanacearum* strains from Africa, South America, North America, and the Caribbean have been sequenced (2). Phylotype I *R. solanacearum* strains from China have already been sequenced (3–5). Though bacterial wilt is severe in India (6), no genomic sequence is available as of yet. We sequenced the *R. solanacearum* strains Rs-09-161 and Rs-10-244, respectively isolated from eggplant (*Solanum melongena*) grown in the west coast region of India and chili (*Capsicum annum*) grown in the Andaman Islands, India. Both strains belong to biovar 3, phylotype I, historically classified as race 1. Endoglucanase (*egl*) gene sequence analysis of *R. solanacearum* isolates collected from India indicated that Rs-09-161 belongs to the major group found within the phylotype I strains and hence represents the *R. solanacearum* population infecting solanaceous vegetables from India. Based on the *egl* gene sequence analysis, Rs-10-244 is the representative *R. solanacearum* isolate from the other subgroup.

Using Illumina HiSeq 2000, we obtained the nucleotide sequences of the Rs-09-161 and Rs-10-244 genomes from a paired-end library with an average insert size of 300 to 500 bp. The trimmed reads were assembled into contigs by using SOAP *de novo* (1.05) and Velvet (1.2.07). Meta-assembly was done using progressiveMauve (2.3.1), with *R. solanacearum* strain GMI1000 as the reference genome. Structural annotation was carried out using EuGene-P (7), with *R. solanacearum* strain GMI1000 as the reference proteome.

The overall G+C content of the Rs-09-161 genome is 66.82%. It contains 5,215 predicted genes, similar to the species complex average of 5,213. The draft genome sequence consists of 5,185 protein-coding genes with a mean gene length of 974 bp and 30 non-protein-coding genes with a mean gene length of 534 bp. The overall G+C content of the Rs-10-244 genome is 66.98%. It contains 5,202 predicted genes. The draft genome sequence consists of 5,171 protein-coding genes with a mean gene length of 969 bp and 31 non-protein-coding genes with a mean gene length of 446 bp. Compared with the published *R. solanacearum* genomes, Rs-09-161 and Rs-10-244 are most closely related to phylotype I strains GMI 1000 and FQY_4.

Further, T3E genes present in these genomes are identified according to the latest nomenclature (2). 71 T3E genes are present in Rs-09-161, which includes two putative candidate T3E genes, and 76 T3E genes are present in Rs-10-244.

### Nucleotide sequence accession numbers.

This whole-genome shotgun project has been deposited at GenBank under the accession numbers JHBO00000000 for strain Rs-09-161 and JHAM00000000 for strain Rs-10-244. The versions described in this paper are the first versions.
